# Advances in Targeted Immunotherapy for Hepatobiliary Cancers

**DOI:** 10.3390/ijms232213961

**Published:** 2022-11-12

**Authors:** Samantha M. Ruff, Alexander H. Shannon, Timothy M. Pawlik

**Affiliations:** Department of Surgery, Division of Surgical Oncology, Wexner Medical Center, The James Comprehensive Cancer Center, The Ohio State University, 395 W. 12th Ave., Suite 670, Columbus, OH 43210, USA

**Keywords:** hepatocellular carcinoma, cholangiocarcinoma, biliary tract cancer, gallbladder cancer, immunotherapy, vaccine, adoptive cell therapy, immune checkpoint inhibitors

## Abstract

Cancer of the hepatobiliary system can be divided into primary liver cancer and biliary tract cancer (BTC), which includes hepatocellular carcinoma (HCC), cholangiocarcinoma (CCA), and gallbladder cancer (GBC). These aggressive cancers often present at an advanced stage or among patients with poorly preserved liver function. The primary treatment for HCC and BTC when diagnosed early is surgical resection, but given the high rate of recurrence and often advanced stage at diagnosis, many patients will require systemic therapy. Unfortunately, even with systemic therapy, long-term survival is poor. The immune system plays an important role in preventing cancer progression. The unique immune environment of the liver and subsequent alterations to the immune microenvironment by tumor cells to create a favorable microenvironment plays a key role in the progression of HCC and BTC. Due to the paucity of effective systemic therapies and distinctive immune environment of the liver, research and clinical trials are investigating the use of immunotherapy in HCC and BTC. This review will focus on current immunotherapies and emerging data for the treatment of HCC and BTC.

## 1. Introduction

Cancer of the hepatobiliary system can be divided into primary liver cancer and biliary tract cancers (BTC), which includes hepatocellular carcinoma (HCC), cholangiocarcinoma (CCA), and gallbladder cancer (GBC). Hepatocellular carcinoma (HCC) is the most common form of liver cancer, accounting for more than 90% of cases [1]. HCC is one of the few cancers with an increasing incidence and mortality [2]. Biliary tract cancers (BTC) are malignancies of the intra or extra hepatic biliary tree or gallbladder. BTC are rare and aggressive malignancies because most are asymptomatic until advanced stages [3].

The primary treatment for HCC and BTC when diagnosed early is surgical resection. As per the Barcelona Clinic Liver Cancer (BCLC), long-term survival in HCC is generally best achieved through liver transplantation, resection of early cancers, or liver directed therapies for small tumors <2–3 cm [4]. For BTC, surgical resection offers potential for curing [5]. Unfortunately, HCC and BTC patients often present with either advanced stage or poorly preserved liver function, which can limit surgical options. In addition, most systemic therapies are limited and ineffective in achieving long term survival [4,6]. Until recently, sorafenib (tyrosine kinase inhibitor) was the first line systemic therapy for advanced HCC. However, the IMbrave150 trial demonstrated that compared to sorafenib, the combination of atezolizumab (PD-L1 inhibitor) and bevacizumab (VEGF inhibitor) conferred a superior survival benefit in patients with advanced HCC [7]. In BTC, the standard of care for adjuvant therapy after resection or advanced disease is currently gemcitabine and cisplatin. However, the prognosis for advanced BTC is dismal with a median survival still being only 8–15 months [8].

**Figure 1:**
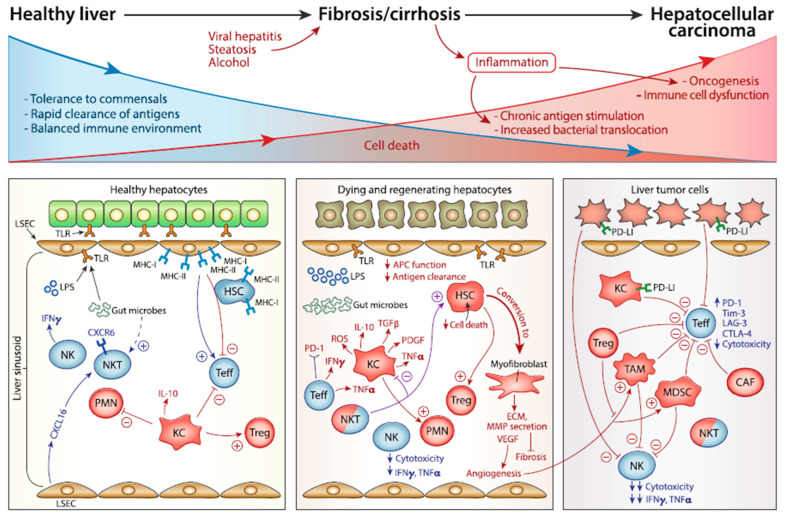
The liver immune microenvironment in the setting of a healthy liver, inflammation, and oncogenesis. This figure was reprinted from reference [12] and information on the license can be found at http://creativecommons.org/licenses/by/4.0/ (accessed on 12 October 2022).

The immune system plays an important role in preventing cancer progression. Alterations to immune surveillance and the adaptive immune system can affect prognosis. Due to its role as a filtration system for toxins, the liver maintains a balance between immune tolerance and activation. Chronic inflammation from hepatitis B, hepatitis C, alcohol damage, and/or non-alcoholic fatty steatosis leads to changes in cell signaling, tissue remodeling, and genetic alterations (Figure 1). These modifications to the microenvironment and disruption of the hepatic immune system facilitate the development of HCC [9,10]. BTC can also arise in the setting of chronic inflammation (e.g., parasitic infections, primary sclerosing cholangitis, primary biliary cirrhosis) or from congenital malformations (e.g., choledochal cysts), but differs from HCC in its microenvironment. CCAs frequently are hypovascular with desmoplastic microenvironments made up of dense collagen stroma, fibroblasts, and fewer tumors associated macrophages/immune cells [11]. The unique immune environment of the liver and subsequent alterations to the immune response by tumor cells to create a favorable microenvironment plays a key role in the progression of HCC and BTC.

Due to the paucity of effective systemic therapies and the distinctive immune environment of the liver, research and clinical trials are investigating the use of immunotherapy in HCC and BTC. Immunotherapy has already reshaped how we treat advanced HCC through updates to the BCLC guidelines [4]. This review will focus on current immunotherapies and emerging data for the treatment of HCC and BTC.

## 2. Immune Checkpoint Inhibitors

**Figure 2:**
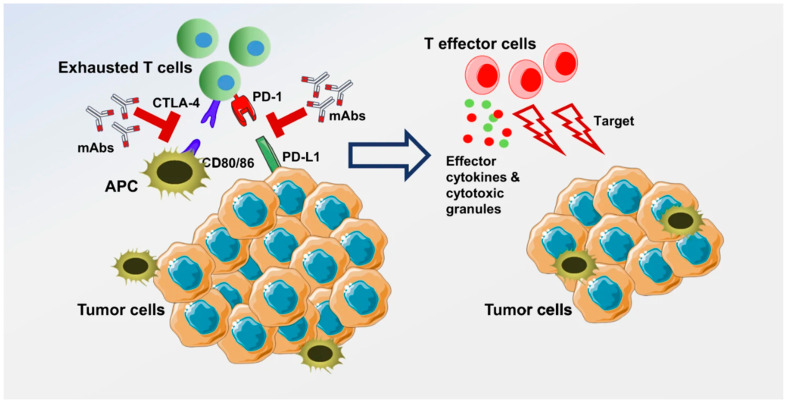
Immune checkpoints expressed on activated T cells lead to inhibition of T cell activation when bound to the ligand on tumor cells/antigen presenting cells. Examples of this include PD-1 and CTLA-4. These interactions are blocked with a monoclonal antibody (immune checkpoint inhibitor), which leads to T cell activation and targeting of tumor cells through release of effector cytokines and cytotoxic granules. This figure was reprinted with from reference [14] and information can be found at http://creativecommons.org/licenses/by/4.0/ (accessed on 12 October 2022).

Immune checkpoint inhibitors (ICIs) have proven to be effective in the treatment of cancer with the benefit of more tolerable side effects than cytotoxic chemotherapy. Immune checkpoints consist of inhibitory and stimulatory immunoreceptors that act as regulators of the immune system. Tumor cells can downregulate surface proteins to prevent activation of stimulatory immunoreceptors or upregulate the expression of proteins that bind inhibitory immunoreceptors to create an immune environment that is immunotolerant of the tumor cells. ICIs aim to block these interactions between tumor and immune cells in order to bolster the antitumor function of immune cells (Figure 2) [13]. Given recent data with the IMbrave150 trial, ICIs encompass a promising area of research [7]. Currently, ICIs have been examined as monotherapy and in combination with other systemic therapies for the treatment of HCC and BTC.

### 2.1. Tremelimumab and Durvalumab in HCC

Tremelimumab is a cytotoxic T-lymphocyte-associated protein 4 (CTLA-4) inhibitor and was the first reported CTLA-4 inhibitor for HCC. CTLA-4 is a receptor on the surface of T cells that competes with CD28 to bind B7 ligands on antigen presenting cells (APCs). When CD28 binds B7, it acts as a co-stimulatory signal for T cells. However, CTLA-4 competes with CD28 to bind B7 and sequester it. Subsequently, B7 is unavailable to bind CD28 to activate T cells [15]. In a phase 2 trial of 20 patients, tremelimumab demonstrated a partial response in 17.6% of patients with HCC. However, the partial response in these three patients only lasted for 3.6, 9.2, and 15.8 months. While this study demonstrated that tremelimumab had an acceptable safety profile, it had a low objective response rate. As a result, it was postulated that combination therapy may be more effective [16].

In response, a randomized phase 2 trial studying tremelimumab and durvalumab (PD-L1 inhibitor) in 332 patients with advanced HCC tested different combinations of the two ICIs. Patients were assigned to receive durvalumab (PD-L1 inhibitor) monotherapy, tremelimumab monotherapy, combination tremelimumab (75 mg) and durvalumab (1500 mg) every four weeks, or a single priming dose of tremelimumab (300 mg) with durvalumab (1500 mg) every four weeks. All mono and dual therapies were safe with acceptable toxicity profiles. Each of the four treatment arms resulted in a durable response, but the tremelimumab priming dose (300 mg) with durvalumab (1500 mg every four weeks) demonstrated the greatest efficacy with an objective response rate of 24% and median overall survival of 18 months. In addition to increased efficacy, by using a priming dose of tremelimumab with durvalumab, the toxicity typically seen with repeated dosing was minimized [17]. In the HIMALAYA phase 3 trial, 1171 patients were assigned to STRIDE combination therapy (tremelimumab (300 mg once) with durvalumab (1500 mg every 4 weeks)), durvalumab monotherapy, or sorafenib. Patients who received STRIDE therapy had improved overall survival compared to sorafenib. The trial was not designed to compare STRIDE and durvalumab monotherapy, but based on the data the authors suspect that tremelimumab may add a survival benefit to durvalumab over time [18].

### 2.2. Tremelimumab and Durvalumab in BTC

Given the rarity of CCA and GBC, clinical trials traditionally group intrahepatic CCA, extrahepatic CCA, and GBC together for the purposes of patient accrual. There are two notable trials evaluating the use of tremelimumab in BTC. The first divided 128 patients with BTC (80 with CCA, 30 with GBC, and 14 with ampullary carcinoma) among three treatment cohorts: gemcitabine/cisplatin, followed by durvalumab and tremelimumab, durvalumab, tremelimumab, and gemcitabine/cisplatin combination therapy, and durvalumab and gemcitabine/cisplatin combination therapy. This study was not designed to evaluate survival and tumor response, but to assess the different dosing regimens of durvalumab and tremelimumab with chemotherapy in patients with BTC. All three combinations demonstrated promising efficacy and acceptable safety profiles. This trial served as the basis for the TOPAZ-1 trial [19]. The recently published TOPAZ-1 trial evaluated durvalumab versus placebo with gemcitabine/cisplatin in 685 patients with advanced BTC. At the interim analysis, durvalumab and gemcitabine/cisplatin significantly improved overall survival (HR 0.8, *p* = 0.021) and progression free survival (HR 0.75, *p* = 0.001). Objective response rate was 26.7% with durvalumab and 18.7% with placebo. There was no difference in grade 3/4 adverse events between the two groups [20].

### 2.3. Nivolumab in HCC

Nivolumab is a programmed death-1 (PD-1) inhibitor and as a result of the Checkmate 040 and Checkmate 459 trials was the first PD-1 inhibitor approved by the FDA in 2017 for treatment of HCC [21,22]. PD-1 is a receptor on T cells, B cells, NK cells, myeloid-derived suppressor cells, and dendritic cells. In response to proinflammatory cytokines, somatic cells express the programmed death ligand-1 (PD-L1) that binds PD-1 and suppresses T-cell migration, proliferation, and secretion of cytotoxins. Tumor cells can take advantage of this pathway by expressing PD-L1 that binds PD-1 on tumor infiltrating lymphocytes inhibiting them, thereby allowing for immune evasion [23]. Interestingly, patients with chronically inflamed livers overexpress PD-1 and PD-L1 [24,25].

The Checkmate 459 trial compared nivolumab with sorafenib among patients with advanced HCC. Nivolumab had a lower rate of grade 3/4 adverse events compared with sorafenib, but there was no statistically significant difference in overall survival. However, the results may be biased since patients who progressed on sorafenib crossed over to the nivolumab cohort and the study utilized an intention to treat analysis [22].

ICIs are increasingly being considered for neoadjuvant or adjuvant therapy in patients with resectable HCC. Surgical resection, ablation, and/or transplantation are generally associated with a 5-year survival in the range of 50–80% [4]. These treatment modalities are not always curative because the microenvironment and immune landscape that promoted the initial HCC is still present and can result in recurrence of disease. Neoadjuvant therapy can take advantage of tumor antigens in the in situ HCC and allow for expansion of naturally occurring tumor specific T lymphocytes. Fifteen patients with high-risk HCC (multinodular disease, portal vein invasion, infiltrative disease, tumor >10 cm) were treated with nivolumab and carbozantinib (tyrosine kinase inhibitor) in a phase 1 trial. Of the 12 patients who underwent surgical resection, four had a >90% pathologic response and one had a complete pathologic response [26]. In a phase 2 trial, nivolumab or combination nivolumab/ipilimumab (CTLA-4 inhibitor) was administered to 27 patients with resectable HCC as neoadjuvant therapy and for 2 years post-resection as adjuvant immunotherapy. Thirty percent of patients had a pathologic response, and no patient had a recurrence at two years post-resection [27]. There are ongoing trials investigating neoadjuvant carbozantinib and nivolumab (NCT03299946), neoadjuvant ipilimumab and nivolumab (NCT03222076, NCT03682276), and adjuvant therapy with nivolumab (CheckMate 9DX NCT03383458).

### 2.4. Nivolumab in BTC

Nivolumab has also been studied in BTC. A phase 2 trial investigated nivolumab among 54 patients with unresectable or metastatic CCA or GBC who had failed at least one other systemic therapy. This study demonstrated that nivolumab was well tolerated with an investigator assessed objective response rate of 22% and a central independent review objective response rate of 11%. In other cancers, PD-1 and PD-L1 inhibitors are commonly most effective in patients with mismatch repair deficiency (MMR-d) or microsatellite instability high (MSI-high) tumors. However, MMR-d or MSI-high is very rare and seen in only 1–2% of patients with CCA. Interestingly, all patients who responded to treatment had MMR-proficient tumors, not MMR-d as would be expected. This likely means that other biomarkers need to be identified for BTC to aid in selecting the most appropriate immunotherapy [28].

Nivolumab was evaluated in a phase 1 trial in Japan in 30 patients with BTC. Chemo naïve patients received nivolumab and cisplatin/gemcitabine and patients who were intolerant/refractory to chemotherapy received nivolumab monotherapy. In the combined therapy cohort, median overall survival was 15.4 months and in the monotherapy cohort median overall survival was 5.2 months [29]. A recent study evaluated the use of nivolumab and gemcitabine/S-1 therapy in 48 patients and demonstrated an objective response rate of 45.9% with a median overall survival of 19.2 months. This study noted that 28.9% of patients harbored loss of function mutations in chromatin remodeling genes and that these patients had a significantly longer progression free and overall survival. The data suggested that loss of function mutations may be a potential biomarker for BTCs [30].

There are two trials that evaluated the combination of ipilimumab (CTLA-4 inhibitor) and nivolumab. The first phase 2 trial evaluated nivolumab/ipilimumab combination therapy in 39 patients with BTC. There was objective response rate of 23% and a disease control rate of 44% [31]. The second trial compared nivolumab and gemcitabine/cisplatin with nivolumab/ipilimumab combination therapy among 75 patients with BTC and had 6-month progression free survival as the primary endpoint. Median overall survival was 10.6 and 8.2 months among patients treated with nivolumab plus gemcitabine/cisplatin versus nivolumab/ipilimumab, respectively. Unfortunately, the addition of nivolumab did not improve 6-month progression free survival [32]. These trials demonstrated an objective response rate of around 20–30%. Identifying the tumor and microenvironment specific factors responsible for these responses will likely be key to choosing an immunotherapy that will yield the greatest clinical benefit.

### 2.5. Pembrolizumab in HCC

Pembrolizumab is a PD-1 inhibitor that has demonstrated efficacy in several cancers. The Keynote-224 trial studied the use of pembrolizumab among patients with HCC who progressed on or were unable to tolerate sorafenib. In this study, 17% of patients had a partial or complete response, 44% had stable disease, and 33% had progressive disease. Pembrolizumab was both safe (24% of patients had a grade 3 adverse event and only 1% had a grade 4 adverse event) and efficacious. As a result of this trial, pembrolizumab was approved by the FDA in 2018 for use in advanced HCC [33].

Despite the early success of the Keynote-224 trial, the Keynote-240 trial that compared pembrolizumab to placebo among 413 randomized patients who progressed on sorafenib failed to demonstrate a difference in overall survival (13.9 months in pembrolizumab cohort versus 10.6 months in placebo cohort). The pembrolizumab cohort did demonstrate an objective response rate of 18.3% versus 4.4% in the placebo cohort. Even though the pre-specified criteria for statistical significance were not met in this trial, both the Keynote-224 and Keynote-240 trial demonstrated that pembrolizumab has some antitumor activity [34]. Despite this, many European societies still do not endorse pembrolizumab given the results of the Keynote-240 trial [35]. Similar to other nivolumab, pembrolizumab is currently being investigated for use as neoadjuvant or adjuvant therapy in resectable HCC (NCT03337841, KEYNOTE-937 NCI03867084).

### 2.6. Pembrolizumab in BTC

A retrospective review of 75 patients with BTC who received PD-1 inhibitors (pembrolizumab, nivolumab, sintilimab, toripalimab) and chemotherapy was compared with 59 patients who only received chemotherapy. Patients who received anti-PD-1 therapy and chemotherapy had a longer progression free survival (5.8 months compared to 3.2 months in the chemotherapy alone cohort, *p* = 0.0004). However, there was no significant difference between objective response rate and disease control rate between the groups [36].

A specific area where pembrolizumab has gained traction is for tumors with microsatellite instability (MSI-high). The Keynote-158 trial treated 233 patients with MSI-high tumors with pembrolizumab who previously progressed on standard therapy of gemcitabine-cisplatin. Within the CCA cohort (22 patients), two patients had a complete response and seven had a partial response. The objective response rate was 40.9% and median overall survival was 24.3 months [37]. In a proof-of-concept study, a cohort of 86 patients with solid tumors were treated with pembrolizumab. Among these patients, four had CCA. One of these patients had a complete response and one had stable disease 12 weeks after initiating therapy [38]. Eleven patients with biliary tract cancers (eight with CCA, three with GBC) were enrolled in a phase 2 trial studying the combination of pembrolizumab with capecitabine and oxaliplatin. Partial response was seen in 17.3% of patients and stable disease was seen in 54% of patients. Median progression free survival was 4.1 months [39].

There are currently ongoing trials for pembrolizumab in BTC. Yin et al. evaluated the safety and efficacy of pembrolizumab and olaparib in 12 patients with advanced CCA. Partial response was seen in one patient, stable disease in four patients, and progressive disease in seven patients. Interim results of this trial indicate that the combination of olaparib and pembrolizumab is safe [40]. The Keynote-966 trial is a currently ongoing randomized, double-blind, phase 3 trial comparing pembrolizumab with gemcitabine/cisplatin versus placebo with gemcitabine/cisplatin in patients with untreated BTC (NCT04003636).

### 2.7. Atezolizumab in HCC

Atezolizumab, a PD-L1 inhibitor, is perhaps the most successful ICI for patients with advanced HCC. The IMbrave150 trial evaluated the combination of atezolizumab and bevacizumab (VEGF inhibitor) compared with sorafenib among 501 randomized patients with advanced HCC and preserved liver function. The IMbrave150 study demonstrated that this combination was associated with improved overall survival (67.2% at 12 months versus 54.6% in the sorafenib cohort) and progression free survival (6.8 months versus 4.3 months in the sorafenib cohort). These data have gone onto inform the treatment of HCC and have resulted in a change to the BCLC guidelines. Of note, the incidence of adverse events was comparable between the two groups. The most serious potential side effect of atezolizumab was upper gastrointestinal bleeding; as such, patients are required to have an upper endoscopy to evaluate for esophageal varices prior to treatment. Given that this trial only included patients with preserved liver function, is unclear if this combination therapy is safe for patients with poor liver function [7]. An update to the IMbrave150 trial noted that combination therapy continued to demonstrate a clinically meaningful survival benefit and consistent safety profile [41]. Based on these results, atezolizumab-bevacizumab was approved by the FDA. According to the BCLC and American Society of Clinical Oncology (ASCO) guidelines, atezolizumab-bevacizumab is now the first line treatment recommendation for patients with advanced HCC [4,42]. There is also an ongoing trial to evaluate if adjuvant atezolizumab and bevacizumab compared with active surveillance is beneficial in patients with resected or ablated HCC (IMbrave050 NCT04102098).

### 2.8. Atezolizumab in BTC

A phase 2 trial randomized 77 patients with BTC to receive atezolizumab monotherapy versus atezolizumab and cobimetinib (MEK inhibitor). The disease control rate was 46.7% versus 30.6% among patients treated with combination therapy versus atezolizumab monotherapy, respectively. There was no difference in overall survival between the two groups [43]. The IMbrave151 trial is an ongoing randomized, double blind, multicenter study comparing atezolizumab, gemcitabine/cisplatin, and bevacizumab versus atezolizumab, gemcitabine/cisplatin, and placebo for patients with advanced BTC (NCT04677504).

### 2.9. Other ICIs under Investigation for HCC

PD-1/PD-L1 and CTLA-4 are the two main ICI targets for the treatment of HCC. However, these therapies are only successful in a fraction of patients with advanced disease. As a result, there is interest in identifying identify novel immune targets for drug development. Given the heterogeneity of HCC tumor antigens within a single tumor, among different tumors in the same patient, and more broadly among patients with HCC, there are an infinite number of potential targets. T cell immunoglobulin mucin-3 (TIM-3), lymphocyte activation gene-3 (LAG-3), and B and T lymphocyte attenuator (BTLA) are some of the more promising targets currently being studied in ongoing trials [44,45,46,47,48,49,50]. The combination of cobolimab (TIM-3 inhibitor) and dostarlimab (PD-1 inhibitor) is currently being evaluated in a phase 2 trials (NCT03680508). Several trials are ongoing with relatlimab (LAG-3 inhibitor) to determine safety/efficacy in patients with HCC (NCT04567615, NCT05337137, NCT04658147).

## 3. Adoptive Cell Therapy

Adoptive cell therapy involves harvesting tumor infiltrating immune cells from the patient and expanding them in the ex vivo setting. The harvested cells can also be genetically engineered for specific targets prior to expansion. After expansion, the cells are infused back into the patient.

### 3.1. Tumor Infiltrating Lymphocytes (TIL) in HCC

Tumor infiltrating lymphocytes (TIL) are harvested immune cells that are selected based on their ability to recognize tumor cells, expanded, and then infused back into the patient with a T cell activating cytokine (IL-2). Prior to infusion the patient undergoes lymphodepletion with cydarabine/fludarabine. TIL is able to recognize multiple tumor antigens and, in theory, be more effective at targeting and destroying tumor cells. In a phase 1 trial, 15 patients with HCC had TIL harvested and generated after surgical resection. No grade 3/4 adverse events were recorded and at a median follow up time of 14 months, all 15 patients were alive. Among these patients, 12 had no recurrent disease [51]. An ongoing clinical trial investigating the safety and efficacy of TIL in patients with recurrent primary HCC is currently ongoing (NCT04538313).

### 3.2. Tumor-Infiltrating Lymphocytes in BTC

TIL is an area of evolving research in the treatment of BTC [52,53]. BTC, including CCA, has a distinctive and complex microenvironment that contributes to immunosuppression and thereby propagation of tumor cells. CCA can be divided into two groups based on the presence or absence of immune cell infiltration within the tumor. Tumors with immune cell infiltration are generally more responsive to therapy [54].

Current literature investigating potential TILs-related immunotherapy for CCA is limited to animal models and cell culture experiments. A study by Diggs et al. treated mice with combined anti-CD40/PD-1 agents and noted impaired CCA cell growth, prolonged mouse survival, and enhanced activation of CD4+/CD8+ T cells and natural killer (NK) cells [55]. Pan et al. treated mice with DNA vaccination targeting CTLA-4/PD-L1, which triggered production of antibodies and suppressed CCA growth [56]. In vitro models have combined cytotoxic T-lymphocytes with gemcitabine or cytokine activated killer cells with cetuximab, which promoted cancer cell death and enhanced cytotoxicity, respectively [57,58]. There is one ongoing trial for TIL in BTC (NCT03801083).

### 3.3. Chimeric Antigen Receptor T Cell (CAR-T Cell) in HCC

Chimeric antigen receptor T-cell (CAR-T cell) therapy harvest T cells from the patient and uses genetic engineering to target specific cancer related antigens. Once infused back into the patient, these CAR-T cells target and bind the tumor antigens, leading to their activation and cytotoxicity. By targeting tumor specific antigens that are minimally expressed in healthy tissue, CAR-T cells can provide an optimal clinical effect with less side effects. Many potential targets have been studied in HCC, including AFP, GPC-3, MAGE, NY-ESO-1, hTERT, NKG2DL, EpCAM, CD133, CD147, and MUC1 [59].

A CAR-T cell targeting GPC-3 (a proteoglycan overexpressed in HCC that activates the Wnt signaling pathway to promote the development of HCC) was evaluated in a trial of 13 patients. The therapy was safe and there was an objective response in two patients [60]. Multiple trials are still ongoing for GPC-3 CAR-T cells [61]. CD133 targeted CAR-T cells have had some success in clinical trials. A phase 1 trial of 23 patients with advanced HCC and CD133 positive tumors demonstrated that repeated cell infusions correlated with longer periods of disease stability. In this study, three patients achieved remission and fourteen patients had stable disease [62]. In a second trial, CD133 targeted CAR-T cells were administered to 21 patients and demonstrated a 6 month disease control rate of 43% [63]. Ongoing trials include one with MUC1 targeted CAR-T cells (NCT02587689) and another with EpCAM targeted CAR-T cells (NCT02729493). Current research with CAR-T cell therapy is also focusing on creating CAR-T cells that can target multiple antigens to improve their efficacy [59].

### 3.4. CAR-T Cell in BTC

While many targets have been identified for HCC, effective targets for BTC remain elusive. One potential target for CCA is Mucin 1 (MUC1), which is highly expressed in these tumors and associated with poor prognosis and survival. Suimon et al. created a fourth generation CAR-T that contained anti-MUC1 domains and assessed their activity on CCA cells. The data demonstrated disruption and cytotoxic effects on cancer cells, suggesting promise for MUC1 as a treatment target for CCA [64]. Another promising target is Integrin αvβ6, which is upregulated in CCA, but not in surrounding epithelial tissues. High expression of Integrin αvβ6 in tumors is associated with shorter survival time. In vitro study have demonstrated that targeting this antigen produced high levels of cytotoxicity in tumor cells [65].

There are a few ongoing clinical trials evaluating CAR-T therapy in CCA. In a phase 1 trial, Guo et al. targeted epidermal growth factor receptor (EGFR), a transmembrane tyrosine kinase protein that is expressed in BTC, pancreatic, breast, and ovarian cancers. The investigators assessed 19 patients with advanced BTC (14 with CCA, 5 with GBC) who were pre-treated with paclitaxel and cyclophosphamide followed by EGFR targeted CAR-T therapy. Response was modest with one patient achieving complete response, ten patients had stable disease, and six patients progressed [66].

HER2 overexpression is well established in many cancers, most notably in breast, ovarian, and endometrial. HER2 overexpression is also present in 3–19% of patients with BTC, more frequently in GBC (16%) than in extrahepatic (11%) or intrahepatic (3%) CCA [67]. Feng et al. looked at HER2, an epidermal growth factor receptor, as another potential target. In a phase I clinical trial, Feng et al. treated eleven patients with advanced HER2 positive BTC with paclitaxel and cyclophosphamide followed by HER2 targeted CAR-T cells. Unfortunately, response was modest with one patient achieving a partial response, five had stable disease, and five patients progressed [68]. Additional targets that are currently under investigation for BTC are mesothelin, CD133, claudin 18.2, and prostate stem cell antigen [69].

## 4. Vaccine Therapy

### 4.1. Vaccine Therapy in HCC

Vaccine therapy is potential means to take advantage of the heterogeneity of HCC and the liver immune tumor microenvironment. Vaccines stimulate a T cell response by delivering antigens or dendritic cells with antigens. Vaccine approaches may target peptides known to be present in HCC (e.g., AFP, GPC-3, MAGE-1, NY-ESO-1, SSX-2, and hTERT) or can be personalized for individual patients by targeting neoantigens. Neoantigens are unique protein sequences that form mutations in tumor cells and are specific to the patient and tumor [70]. A phase 1/2 vaccine trial using dendritic cells with AFP, GPC-3, and MAGE-1 antigens resulted in disease stabilization in 60% of patients versus the control group [71]. In a different phase 1/2 trial studied the safety and efficacy of the HepaVac-101 vaccine that targeted multiple antigens in 22 patients with early to intermediate stage HCC and suitable HLA haplotypes. The vaccine had an acceptable safety profile and had an immune response against HLA class I and II tumor peptides in 37% and 53% of patients, respectively [72]. Clinical trials evaluating vaccine therapy as either monotherapy or in combination with ICIs are currently ongoing, but most are not published or have yielded negative clinical results.

### 4.2. Vaccine Therapy in BTC

Vaccines for BTC are another area of evolving research. Like HCC, the liver immune tumor microenvironment lends itself to the creation of vaccines to target BTC, although development of an efficacious vaccine has been challenging. Rat models have suggested the potential of a DNA vaccine that targets CTLA-4 and PD-1 in CCA [56]. Huang et al. aimed to identify potential antigens of CCA in vitro to develop an mRNA vaccine [73]. Three tumor antigens, CD247, FCGR1A, and TRRAP, were identified as potential targets for mRNA vaccine development.

Clinical trials investigating BTC vaccines have been performed with mixed results. One phase I trial examined Wilm’s tumor 1 (WT1) peptide vaccine in combination with gemcitabine and cisplatin to treat patients with advanced pancreatic and biliary tumors. However, there was no convincing clinical benefit [74,75]. Another phase I trial using a multi-peptide vaccine (KIF20A) demonstrated a median overall survival of 9.7 months and a targeted immune response in all patients. Five of the nine patients had stable disease [76]. A more recent phase 2 trial from Japan assessed the immune response and clinical benefit of OCV-C01, a three peptide vaccine that specifically targets vascular endothelial growth factor receptors (VEGFR) 1 and 2. VEGFR 1 and 2 are tyrosine kinase receptors associated with proliferation of vascular endothelial cells in tumors. The third peptide in the vaccine was KIF20A, which had shown some benefit in previous phase 1 trials. Four of the six patients elicited an immune response to the vaccine that may have contributed to survival [77]. There are ongoing clinical trials in this area, many of which are not yet concluded or published (Table 1) [69].

## 5. Challenges of Immunotherapy

Unfortunately, not all patients with liver cancer respond to immunotherapy. Heterogeneity of tumor antigens within a single tumor, between tumors in the same patient, and across tumors in different patients are all obstacles for immunotherapy. Tumor heterogeneity may be due to the multiple mechanisms that can give rise to liver cancer and the complexity of the immune microenvironment. Tumor heterogeneity results in many possible targets for ICIs and adoptive cell therapy, yet there is no good way to identify which patients will respond. The success of clinical trials focused on multi-modal treatment (ICIs with VEGF inhibitors or CAR-T cells/TIL that target multiple tumor antigens) has demonstrated that identification of patient subsets will be a key strategy moving forward in the treatment of HCC and BTC. Continued success will rely on continuing to discover new targets, collaborative work among high volume centers to aid in trial accrual, and focused work on identifying patient/tumor factors that correlate with successful immunotherapy treatment.

Another obstacle is the delivery of ICIs, TIL, and CAR-T cells to the tumor. For example, because HCC often consists of a dense fibrotic matrix, it can be difficult for ICIs and CAR-T cells to access the tumor. The combination of ICIs with VEGF inhibitors to alter the tumor vasculature and the work to design CAR-T cells, which also express enzymes that can degrade the extracellular matrix, may help improve delivery [79]. Some preliminary work has been done to delivery adoptive cell therapy directly to the tumor through hepatic artery infusion rather than peripheral infusion [80,81,82].

## 6. Conclusions

HCC, CCA, and GBC are rare, aggressive tumors that often present at an advanced stage and have few systemic therapy options. Alterations to the unique immune microenvironment of the liver plays an important role in cancer development and progression. As a result, current research and clinical trials are focused on identifying immunotherapy that is effective for HCC and BTC. These efforts include the use of immune checkpoint inhibitors, adoptive cell therapy, and vaccine development. Despite promising results in the laboratory, only about a quarter of patients respond to immunotherapy in these clinical trials, whether that is measured through survival or objective response rate. The heterogeneity of HCC and BTC tumor antigens and the resulting innumerable potential targets is likely related to the mixed results with this therapeutic approach. More success has been achieved with combination therapies (i.e., often an immune checkpoint inhibitor with a targeted monoclonal antibody or cytotoxic chemotherapy). Future efforts will need to identify why certain subsets of patients respond to immunotherapy. By leveraging this information, more personalized treatment plans can be developed to maximize clinical response with first- or second-line therapy for patients with HCC and BTC.

## Figures and Tables

**Table 1:** Ongoing clinical trials of cancer vaccines and adoptive cell therapy in cholangiocarcinoma. Reprinted/adapted with permission from Ref [78] 2022, Elsevier Science & Technology Journals.

Identifier	Study Arm	Phase	Enrollment	Primary Endpoint	Stage	Study Start Date
NCT0304182	Oral therapeutic vaccine V3-X	1/2	20	Changes in CA19.9	Unknown	20 February 2017
NCT03633773	MUC-1 CART cell immunotherapy cytokine induced killer cells	1/2	9	Disease control rate	Recruiting	1 July 2018
NCT01868490	Cytokine induced killer cells	1/2	13	MRI scan for monitoring of tumor size and CIK cell-homing; Fluorescence activated cell sorting	Unknown	17 April 2009
NCT04951141	Anti-GPC3 CAR T	1	10	Adverse events	Recruiting	1 January 2019
NCT03801083	Tumor infiltrating lymphocytes	2	59	Objective Response Rate	Recruiting	19 February 2019
NCT03942328	External beam radiation therapy, autologous dendritic cells, and Prevnar	1	26	Incidence of significant toxicity	Recruiting	17 May 2019
NCT03907852	Gavo-cel, fludarabine, cyclophosphamide; Gavo-cel, fludarabine, cyclophosphamide, anti-PD1	1/2	70	Safety	Recruiting	15 April 2019
NCT4853017	ELI-002 2P Amph-CpG-7909 admixed with Amph modified KRAS peptides	1	18	Safety	Recruiting	4 October 2021
NCT05194735	TCR-T Cell Drug Product; TCR-T Cell Drug Product with Aldesleukin (IL-2)	1/2	180	Safety	Recruiting	February 2022
NCT04660929	CT-0508	1	18	Safety	Recruiting	2 February 2021

## Data Availability

Not applicable.

## Abbreviations

PD-L1	programmed death ligand 1
PD-1	programmed death 1
VEGF	vascular endothelial growth factor
CTLA-4	cytotoxic T-lymphocyte-associated protein 4
MMR	mismatch repair
IL-2	interleukin 2
TIL	tumor infiltrating lymphocytes
BTC	biliary tract cancers
HCC	hepatocellular carcinoma
CCA	cholangiocarcinoma
GBC	gallbladder cancer
AFP	alpha fetoprotein
GPC-3	glypican-3
MAGE	melanoma associated antigen
NY-ESO-1	New York esophageal squamous cell carcinoma 1
hTERT	human telomerase reverse transcriptase
NKG2DL	natural killer group 2, member D ligand
EpCAM	epithelial cellular adhesion molecule
CD133	prominin-1
CD147	cluster of differentiation 147
MUC1	mucin 1
EGFR	epidermal growth factor receptor
Her2	human epidermal growth factor receptor
SSX-2	synovial sarcoma X
APC	antigen presenting cell
NK	natural killer
CAR-T	chimeric antigen receptor T cell
